# Surgical venous-to–pulmonary artery temporary right ventricular support after durable left ventricular assist device implantation: A single-center experience

**DOI:** 10.1016/j.jhlto.2026.100682

**Published:** 2026-09-10

**Authors:** Matthan Tharakan, Luis M. Quiroz, Steven Stroud, Alexander Hajduczok, Adam Betz, Daniel Hopkins, Rachel Posey, Michaela Sangillo, Ajit K. Tharakan

**Affiliations:** a Department of Surgery, University of Oklahoma Health Sciences Center, Oklahoma City, Oklahoma; b Department of Cardiology, Oklahoma Heart Institute, Tulsa, Oklahoma; c Department of Cardiovascular Critical Care, Oklahoma Heart Institute, Tulsa, Oklahoma; d Department of Cardiothoracic Surgery, Oklahoma Heart Institute, Tulsa, Oklahoma

**Keywords:** left ventricular assist device (LVAD), right heart failure, venous-to-pulmonary artery support, advanced heart failure, device implant

## Abstract

**Background:**

Postoperative right heart failure (RHF) remains a major complication after durable left ventricular assist device (LVAD) implantation and is associated with substantial morbidity, mortality, and resource utilization. Temporary right-sided mechanical circulatory support strategies are increasingly used for severe RHF, but comparative real-world data remain limited. We evaluated the incidence of postoperative RHF after durable LVAD implantation, its association with early outcomes, and institutional RHF treatment patterns.

**Methods:**

We performed a retrospective single-center cohort study of 79 consecutive adult patients who underwent durable LVAD implantation at Oklahoma Heart Institute between January 2018 and April 2026. Patients were categorized by the presence or absence of postoperative RHF. The primary analysis compared early postoperative outcomes between RHF and non-RHF groups. Continuous variables were compared using the Wilcoxon rank-sum test and categorical variables using Fisher’s exact test.

**Results:**

Postoperative RHF occurred in 25 of 79 patients (32%). Baseline demographic and clinical characteristics did not differ significantly between RHF and non-RHF groups. Compared with patients without RHF, those with RHF had longer pre-implant length of stay (14 vs 10 days, *p* = 0.03), intensive care unit length of stay (21 vs 9 days, *p* < 0.001), total hospital length of stay (44 vs 34 days, *p* = 0.01), and post-implant length of stay (25 vs 21 days, *p* = 0.1). Follow-up status did not differ significantly between groups (*p* = 0.7). Among the 25 patients with RHF who required mechanical support, treatment strategies included T-VAD in 19 patients, ProtekDuo in 4 patients, and venoarterial extracorporeal membrane oxygenation in 2 patients. Observed follow-up status varied by modality, with 18 of 19 patients (95%) treated with T-VAD alive at last follow-up. Given the small, clinically heterogeneous comparator group, formal between-group comparison was not performed.

**Conclusions:**

Postoperative RHF occurred in one-third of patients after durable LVAD implantation and was associated with significantly greater perioperative resource utilization. T-VAD was the predominant temporary RV support strategy at our institution and was feasible, with favorable observed survival in this descriptive single-center experience. Differences across support modalities likely reflect distinct clinical phenotypes and indications rather than a device effect and should not be interpreted as comparative evidence.

## Background

Durable left ventricular assist device (LVAD) therapy has become an established treatment for patients with advanced heart failure, but postoperative right heart failure (RHF) remains a major complication. The incidence of acute RHF varies from 10% to 40%, and when severe RHF requires mechanical circulatory support, in-hospital mortality can reach nearly 50%.^1^, ^2^ RHF is also associated with prolonged hospitalization, rehospitalization, mortality, and adverse events including stroke and gastrointestinal bleeding.^2^, ^3^

Patients who develop RHF after LVAD implantation often require prolonged intensive care unit (ICU) care, extended hospitalization, and escalation to temporary right-sided mechanical circulatory support.^3^, ^4^ Management options include surgical right ventricular assist device (RVAD) configurations, percutaneous devices such as ProtekDuo, and venoarterial extracorporeal membrane oxygenation (VA ECMO); however, comparative real-world data remain limited, and support selection is often shaped by institutional practice, timing of RHF recognition, and patient acuity.^1^, ^6^, ^7^, ^8^

We performed a retrospective single-center cohort study of 79 consecutive durable LVAD recipients to evaluate the incidence of postoperative RHF, its association with early clinical outcomes, and institutional RHF treatment patterns.

## Methods

### Study design and patient population

We performed a retrospective single-center cohort study of consecutive adult patients who underwent durable LVAD implantation at Oklahoma Heart Institute, Tulsa, Oklahoma. Patients were identified from an institutional database of LVAD recipients from 2018 to 2026. The study cohort included 79 patients with available implant and follow-up data. Patients were categorized according to the presence or absence of postoperative right heart failure (RHF). Among patients who developed RHF, outcomes were summarized descriptively by temporary support modality. The choice of temporary RV support strategy reflected an evolution in institutional practice rather than a per-patient algorithm. Earlier in the series, refractory postoperative RHF was managed with non–T-VAD strategies (ProtekDuo or VA ECMO); the program subsequently adopted a surgically configured T-VAD approach as the preferred strategy. Support modality was therefore closely linked to era of implantation and accumulating institutional experience.

### Definition of postoperative right heart failure

Postoperative right heart failure (RHF) was defined according to the Mechanical Circulatory Support Academic Research Consortium (MCS-ARC) adverse event definition.^9^ Early acute RHF was defined as the need for temporary or durable right ventricular assist device (RVAD) support, including extracorporeal membrane oxygenation, concomitant with LVAD implantation before leaving the operating room. Early post-implant RHF was defined as the need for temporary or durable RVAD support, including extracorporeal membrane oxygenation, within 30 days after LVAD implantation for any duration. Patients also met RHF criteria if they had failure to wean from inotropic, vasopressor, or inhaled nitric oxide support within 14 days after LVAD implantation, or required initiation of this support within 30 days for at least 14 days, together with the specified clinical or end-organ manifestations. In the present cohort, all patients classified as having postoperative RHF met MCS-ARC criteria through the requirement for temporary RVAD or ECMO support after durable LVAD implantation.

### Operative technique (venous to pulmonary artery RVAD configuration)

**Figure 1:**
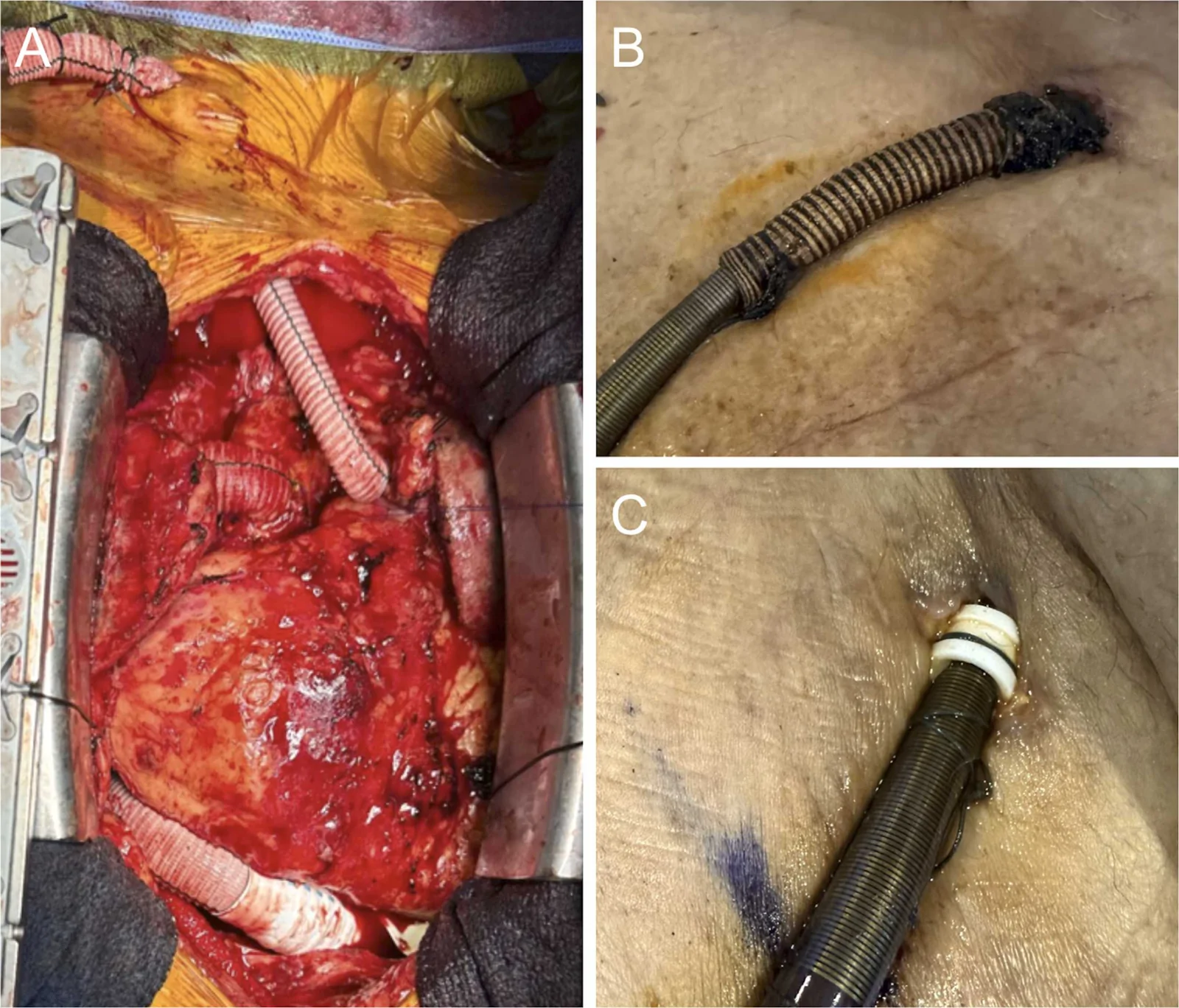
(A) Intraoperative image of a completed HeartMate 3 implant with temporary T-VAD in place. (B) Tunneled 19 french return cannula through an 8 mm gelweave graft sown onto the main PA. (C) Twenty-five french long drainage cannula from the right atrium through the right femoral vein. T-VAD, Tharakan-Ventricular Assist Device.

Temporary venous-to-pulmonary artery support was established during the index LVAD operation. An 8-mm Dacron graft was anastomosed end-to-side to the main pulmonary artery under a partial-occlusion clamp and trimmed to a length avoiding kinking or tension with the lungs inflated. The graft was tunneled subcutaneously to an exit site at the right side of the neck above the clavicle, rather than to the subxiphoid or subcostal region as in previously described graft-based configurations, where a 19-Fr arterial return cannula was secured within it. This configuration, referred to hereafter as the Tharakan-Ventricular Assist Device (T-VAD), keeps the graft exit clear of the abdomen and chest wall, permitting primary sternal closure and unrestricted sitting, transfer, and ambulation during support. Venous drainage was obtained through a percutaneously placed multistage long right femoral venous cannula. Both limbs were connected to a CentriMag Acute Mechanical Circulatory Support Pump (Abbott) circuit, and a Nautilus ECMO Oxygenator (Medtronic) was incorporated in-line when gas exchange support was required. Flow was initiated intraoperatively and titrated according to hemodynamics, right ventricular decompression on transesophageal echocardiography, and LVAD flows (Figure 1A–C).

### Weaning and decannulation

RVAD support was weaned in a stepwise fashion with serial reduction in circuit flow, assessing right ventricular size and function, central venous pressure, and LVAD flows echocardiographically at each step. Patients maintaining hemodynamic stability at low flow without escalation of inotropic support proceeded to decannulation. Decannulation was performed in the operating room or at bedside by withdrawing the cannula, ligating and oversewing the graft, and allowing the retained segment to retract into the mediastinum, avoiding resternotomy. No graft-related complications were observed during follow-up.

### Data collection

Demographic, clinical, perioperative, and outcome data were collected through retrospective review of the institutional dataset. Variables abstracted for analysis included age, sex, race, body mass index (BMI), heart failure etiology, Interagency Registry for Mechanically Assisted Circulatory Support (INTERMACS) profile, ejection fraction at the time of implant, inotrope use at implant, pre-implant mechanical circulatory support, patient status at follow-up, and postoperative complications. Length-of-stay variables collected included pre-implant length of stay, ICU length of stay, total hospital length of stay, and post-implant length of stay. RHF treatment modality was recorded from the dataset and categorized according to the documented support strategy.

The primary outcome was postoperative RHF following durable LVAD implantation. Among patients with postoperative RHF, treatment modality was categorized according to the documented temporary support strategy. Support modalities included T-VAD support, ProtekDuo dual-lumen cannula support, and venoarterial extracorporeal membrane oxygenation (VA ECMO). Outcomes among patients with RHF were summarized descriptively by support modality (T-VAD, ProtekDuo, and VA ECMO). No formal between-group statistical comparison was performed given the small, clinically heterogeneous subgroups.

### Outcomes

The primary outcome of the overall cohort analysis was the association between postoperative RHF and early postoperative outcomes. Outcomes of interest included ICU length of stay, total hospital length of stay, post-implant length of stay, patient status at follow-up, and major postoperative complications including stroke and gastrointestinal bleeding.

### Statistical analysis

Continuous variables were summarized as medians with interquartile ranges (IQRs), and categorical variables were summarized as counts with percentages. Comparisons between patients with and without postoperative RHF were performed using the Wilcoxon rank-sum test for continuous variables and Fisher’s exact test for categorical variables. Kaplan–Meier survival analyses were performed to compare survival following LVAD implantation between patients with and without postoperative RHF and, among patients with RHF, between those treated with T-VAD versus non–T-VAD support. Differences between survival curves were assessed using the log-rank test.

Exploratory logistic regression analyses were performed to assess associations between selected baseline variables and postoperative RHF. Univariable logistic regression models were constructed for clinically relevant covariates, and odds ratios with 95% confidence intervals were estimated. Given the modest sample size and missingness across several variables, multivariable modeling was limited and interpreted as exploratory. Statistical significance was defined as a two-sided *p*-value < 0.05. All analyses were performed in R version 4.5.3 (R Foundation for Statistical Computing, Vienna, Austria).

### Ethical considerations

This study was conducted in accordance with institutional review board requirements for retrospective research using existing clinical data. Patient confidentiality was maintained throughout the study.

## Results

### Cohort characteristics

A total of 79 durable LVAD recipients were included. Median age was 60 years (Q1, Q3: 49, 68), 54 patients (67%) were male, and median BMI was 28 kg/m² (Q1, Q3: 24, 35). INTERMACS profiles were distributed as follows: 19 patients (24%) in profile 1, 25 (32%) in profile 2, 20 (25%) in profile 3, and 15 (19%) in profile 4. Heart failure etiology was ischemic in 31 patients (39%), nonischemic in 46 (58%), and other in 2 (2.5%). Median ejection fraction at implant was 20.0% (Q1, Q3: 15.0, 20.0). At follow-up, 58 patients (73%) were living, 18 (23%) had expired, and 3 (4%) had undergone transplant (Table 1).

**Table 1:** Baseline Characteristics of Overall LVAD Cohort

**Characteristic**	***N* = 79**
Age, years	60 (49, 68)
Sex	
Female	26/79 (33%)
Male	53/79 (67%)
Race/ethnicity	
African American	19/79 (24%)
American Indian/Alaskan Native	8/79 (10%)
Hispanic/Latino	3/79 (3.8%)
White	49/80 (62%)
Body mass index, kg/m²	28 (24, 35)
INTERMACS profile	
INTERMACS 1	19/79 (24%)
INTERMACS 2	25/79 (32%)
INTERMACS 3	20/79 (25%)
INTERMACS 4	15/79 (19%)
Heart failure etiology	
Ischemic	31/79 (39%)
Nonischemic	46/79 (58%)
Other	2/79 (2.5%)
Ejection fraction at implant, %	20.0 (15.0, 20.0)
LVAD device	
HeartMate 2	1/79 (1%)
HeartMate 3	64/79 (81%)
HeartWare Ventricular Assist Device	14/79 (18%)
Inotrope at implant	
Dobutamine	11/79 (14%)
Milrinone	32/79 (41%)
None	36/79 (46%)
Pre-implant mechanical circulatory support	
IABP	1/79 (1%)
Impella	17/79 (22%)
None	61/79 (77%)
Pre-implant length of stay, days	11 (6, 17)
ICU length of stay, days	14 (7, 24)
Total hospital length of stay, days	36 (25, 52)
Post-implant length of stay, days	23 (15, 34)
Compications	
Drive Line Infection	11/79 (14%)
Stroke	9/79 (11%)
Gastrointestinal bleeding	8/79 (10%)
Pump thrombosis	2/79 (3%)
Follow-up status	
Expired	18/79 (23%)
Living	58/79 (73%)
Transplant	3/79 (4%)

IABP, intra-aortic balloon pump; ICU, intensive care unit; INTERMACS, Interagency Registry for Mechanically Assisted Circulatory Support; LVAD, left ventricular assist device.

Values are presented as median (Q1, Q3) or *n/N* (%). Percentages may not sum to 100% because of rounding.

### Postoperative right heart failure

Postoperative RHF occurred in 25 of 79 patients (32%), while 54 patients (68%) did not develop RHF. There were no significant differences between groups with respect to age, sex, race/ethnicity, body mass index, INTERMACS profile, heart failure etiology, ejection fraction at implant, LVAD device, inotrope use, or pre-implant circulatory support (Table 2). Median age was 61 years (Q1, Q3: 43, 67) in the RHF group and 58 years (Q1, Q3: 49, 68) in the non-RHF group (*p* = 0.8). Median body mass index was 29 kg/m² (Q1, Q3: 25, 35) versus 28 kg/m² (Q1, Q3: 24, 34), respectively (*p* = 0.8), and median ejection fraction at implant was 20.0% (Q1, Q3: 13.0, 20.0) versus 20.0% (Q1, Q3: 15.0, 20.0), respectively (*p* = 0.3).

**Table 2:** Comparison of Patients With and Without Postoperative Right Heart Failure

**Characteristic**	**No RHF**	**RHF**	***p*-value**
	N = 54	N = 25	
Age, years	58 (49, 68)	61 (43, 67)	0.8
Sex			0.8
Female	17/54 (31%)	9/25 (36%)	
Male	37/54 (69%)	16/25 (64%)	
Race/ethnicity			0.2
African American	13/54 (24%)	6/25 (24%)	
American Indian/Alaskan Native	3/54 (6%)	5/25 (20%)	
Hispanic/Latino	2/54 (4%)	1/25 (4%)	
White	36/54 (67%)	13/25 (52%)	
Body mass index, kg/m²	28 (24, 34)	29 (25, 35)	0.8
INTERMACS profile			0.7
INTERMACS 1	13/54 (24%)	6 / 25 (24%)	
INTERMACS 2	15/54 (28%)	10 / 25 (40%)	
INTERMACS 3	14/54 (26%)	6 / 25 (24%)	
INTERMACS 4	12/54 (22%)	3/25 (12%)	
Heart failure etiology			0.5
Ischemic	23/54 (43%)	8/25 (32%)	
Nonischemic	30/54 (56%)	16/25 (64%)	
Other	1/54 (2%)	1/26 (4%)	
Ejection fraction at implant, %	20.0 (15.0, 20.0)	20.0 (13.0, 20.0)	0.3
LVAD device			0.2
HeartMate 2	1/54 (2%)	0/25 (0%)	
HeartMate 3	41/54 (76%)	23/25 (92%)	
HeartWare Ventricular Assist Device	12/54 (22%)	2/25 (8%)	
Inotrope at implant			0.082
Dobutamine	7/54 (13%)	4/25 (16%)	
Milrinone	18/54 (33%)	14/25 (56%)	
None	29/54 (54%)	7/25 (28%)	
Pre-implant circulatory support			0.1
IABP	0/54 (0%)	1/25 (4%)	
Impella	9/54 (17%)	8/25 (32%)	
None	45/54 (83%)	16/25 (64%)	
Pre-implant length of stay, days	10 (3, 15)	14 (7, 23)	0.03
ICU length of stay, days	9 (6, 21)	21 (15, 29)	<0.001
Total hospital length of stay, days	34 (22, 49)	44 (34, 55)	0.01
Post-implant length of stay, days	21 (14, 29)	25 (22, 42)	0.1
Compications			
Drive Line Infection	10/54 (19%)	1/25 (4%)	0.2
Stroke	7/54 (13%)	2/25 (8%)	0.7
Gastrointestinal bleeding	7/54 (13%)	1/25 (4%)	0.4
Pump thrombosis	1/54 (2%)	1/25 (4%)	0.5
Follow-up status			0.7
Expired	12/54 (22%)	6/25 (24%)	
Living	39/54 (72%)	19/25 (76%)	
Transplant	3/54 (6%)	0/25 (0%)	

IABP, intra-aortic balloon pump; ICU, intensive care unit; INTERMACS, Interagency Registry for Mechanically Assisted Circulatory Support; LVAD, left ventricular assist device; RHF, right heart failure.

Values are presented as median (Q1, Q3) or *n*/*N* (%). *P*-values were calculated using the Wilcoxon rank-sum test for continuous variables and Fisher’s exact test for categorical variables. Percentages may not sum to 100% because of rounding.

Patients with postoperative RHF had significantly longer pre-implant, ICU, and total hospital lengths of stay. Median pre-implant length of stay was 14 versus 10 days (*p* = 0.03), ICU length of stay was 21 versus 9 days (*p* < 0.001), and total hospital length of stay was 44 versus 34 days (*p* = 0.01) for RHF and non-RHF patients, respectively. Post-implant length of stay was numerically longer in the RHF group but was not statistically significant (25 vs 21 days; *p* = 0.1).

**Figure 2:**
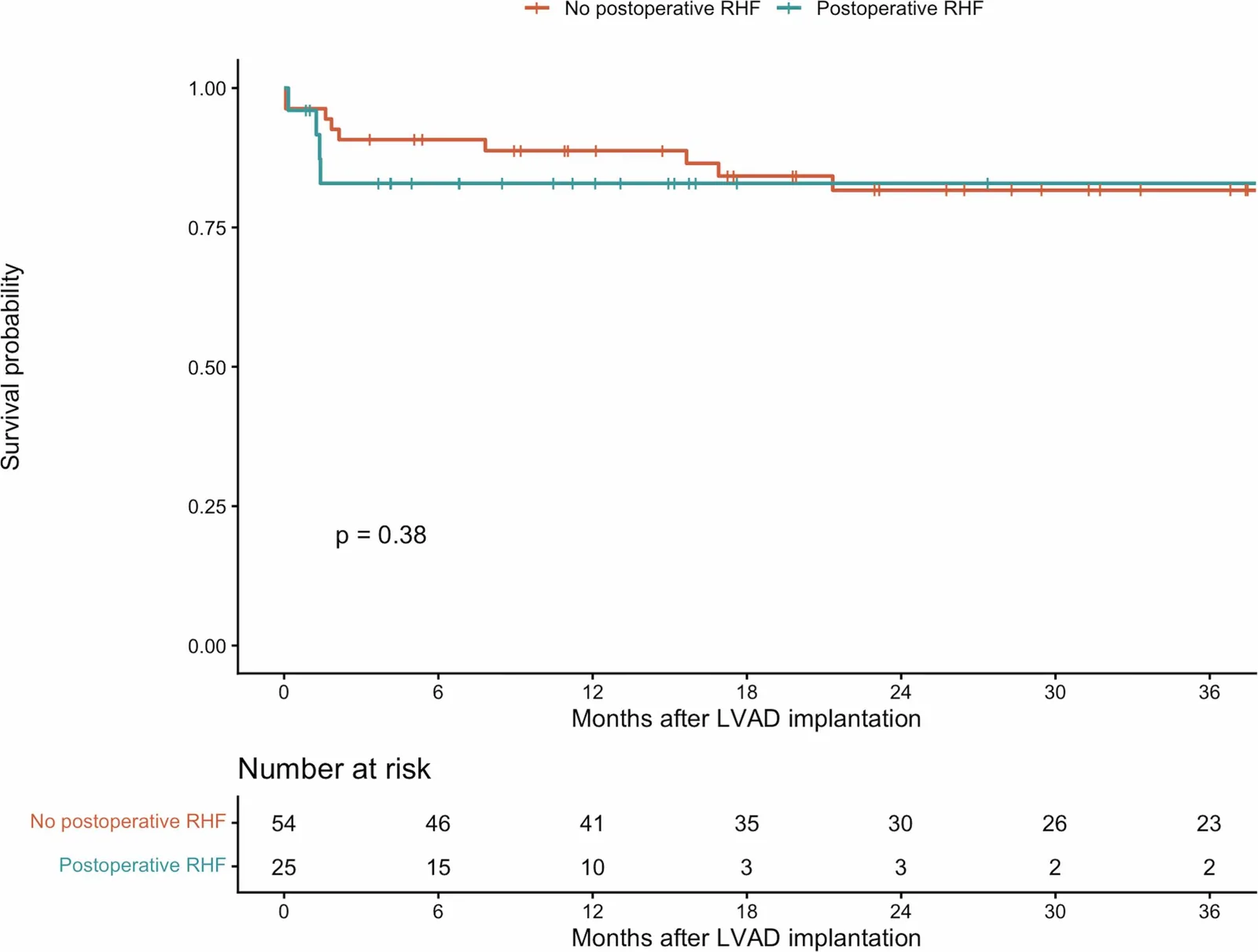
Kaplan–Meier survival analysis following LVAD implantation. Survival stratified by postoperative right heart failure status. Patients with postoperative RHF demonstrated lower survival compared with those without postoperative RHF (log-rank *p* = 0.38). LVAD, left ventricular assist device; RHF, right heart failure.

Patient status at follow-up did not differ significantly between groups (*p* = 0.7). Mortality was similar between groups, occurring in 6 of 25 RHF patients (24%) and 12 of 54 non-RHF patients (22%). There were no statistically significant differences in driveline infection (*p* = 0.2), stroke (*p* = 0.7), gastrointestinal bleeding (*p* = 0.4), or pump thrombosis (*p* = 0.5) between groups (Table 2). Figure 2 shows a Kaplan–Meier analysis demonstrating overall survival was not significantly different between patients with and without postoperative RHF (log-rank *p* = 0.38).

### RHF treatment strategies

Among the 25 patients with postoperative RHF, temporary support consisted of T-VAD in 19 patients, ProtekDuo in 4 patients, and VA ECMO in 2 patients. Median age was 61 years (Q1, Q3: 43, 69) in the T-VAD group, 63 years (Q1, Q3: 51, 65) in the ProtekDuo group, and 46 years (Q1, Q3: 31, 61) in the VA ECMO group. Median ICU length of stay was 18 days (Q1, Q3: 14, 24), 43 days (Q1, Q3: 40, 45), and 16 days (Q1, Q3: 7, 24), respectively, and median total hospital length of stay was 41 days (Q1, Q3: 29, 55), 50 days (Q1, Q3: 44, 71), and 43 days (Q1, Q3: 34, 52), respectively. Median follow-up was 9.5 months, 1.4 months, and 80.7 months, respectively (Table 3).

**Table 3:** Descriptive Outcomes by Temporary Right Heart Failure Support Strategy

**Characteristic**	**T-VAD**	**Protek Duo**	**VA ECMO**
	*N* = 19	*N* = 4	*N* = 2
Age, years	61 (43, 69)	63 (51, 65)	46 (31, 61)
Sex			
Female	7/19 (37%)	2/4 (50%)	0/2 (0%)
Male	12/19 (63%)	2/4 (50%)	2/2 (100%)
Body mass index, kg/m²	28 (22, 31)	35 (29, 41)	30 (18, 42)
INTERMACS profile			
INTERMACS 1	4/19 (21%)	1/4 (25%)	1/2 (50%)
INTERMACS 2	9/19 (47%)	0/4 (0%)	1/2 (50%)
INTERMACS 3	3/19 (16%)	3/4 (75%)	0/2 (0%)
INTERMACS 4	3/19 (16%)	0/4 (0%)	0/2 (0%)
Pre-implant length of stay, days	16 (10, 23)	10 (4, 20)	18 (7, 28)
ICU length of stay, days	18 (14, 24)	43 (40, 45)	16 (7, 24)
Total hospital length of stay, days	41 (29, 55)	50 (44, 71)	43 (34, 52)
Post-implant length of stay, days	22 (20, 31)	43 (40, 52)	26 (24, 27)
Complications			
Drive Line Infection	1/19 (5%)	0/4 (0%)	0/2 (0%)
Stroke	2/19 (11%)	0/4 (0%)	0/2 (50%)
Gastrointestinal bleeding	0/19 (0%)	1/4 (25%)	0/2 (0%)
Reoperation for bleeding	2/19 (11%)	0/4 (0%)	0/2 (0%)
Renal replacement therapy	2/19 (11%)	4/4 (100%)	1/2 (50%)
Pump thrombosis	0/19 (0%)	1/4 (25%)	1/2 (50%)
Discharged on inotropes	0/18 (0%)	0/1 (0%)	0/2 (0%)
Follow-up status			
Expired	1/19 (5%)	3/4 (75%)	2/2 (100%)
Living	18/19 (95%)	1/4 (25%)	0/2 (0%)
Transplant	0/19 (0%)	0/4 (0%)	0/2 (0%)
Discharge Outcomes			
Died during index admission	1/19 (5%)	3/4 (75%)	2/2 (100%)
Survived to hospital discharge	18/19 (95%)	1/4 (25%)	0/2 (0%)
Death after discharge	0/19 (0%)	0/4 (0%)	0/2 (0%)

ICU, intensive care unit; INTERMACS, Interagency Registry for Mechanically Assisted Circulatory Support; RHF, right heart failure; VA ECMO, venoarterial extracorporeal membrane oxygenation.

Values are presented as median (Q1, Q3) or *n*/*N* (%). This table is descriptive only; formal statistical comparisons were not performed because of small subgroup sizes.

### Outcomes by support modality

Outcomes by temporary right ventricular support strategy are summarized descriptively in Table 3. Among the 19 patients managed with T-VAD support, median duration of support was 11 days (Q1, Q3: 8, 15) and an oxygenator was incorporated into the circuit in 6 patients (32%). One patient (5%) died during the index admission and 18 (95%) survived to hospital discharge, with no deaths occurring after discharge. Reoperation for bleeding was required in 2 patients (11%) and renal replacement therapy in 2 patients (11%), and no patient surviving to discharge required continuous inotropic support. Among the 6 patients managed with ProtekDuo or VA ECMO, 1 (17%) survived to hospital discharge; renal replacement therapy was required in all 4 ProtekDuo patients and in 1 VA ECMO patient. Pump thrombosis occurred in 1 ProtekDuo patient (25%) and 1 VA ECMO patient (50%). These groups differed in clinical context: T-VAD was used predominantly as intraoperative or early right ventricular support, whereas ProtekDuo and VA ECMO were used predominantly as postoperative rescue for refractory or combined cardiorespiratory failure.

## Discussion

In this single-center cohort of 79 durable LVAD recipients, postoperative RHF occurred in 33% of patients and was associated with substantially greater perioperative resource utilization, including longer pre-implant, ICU, total hospital, and post-implant length of stay. Among patients who developed RHF, T-VAD was the predominant temporary RV support strategy, used in 19 of 25 patients, and was accompanied by favorable observed survival.

### Post-LVAD RHF and clinical burden

The 32% incidence of RHF in this cohort is consistent with reported rates of acute RHF after LVAD implantation, which range from 10% to 40%.^1^, ^5^ Although standardized definitions have been proposed, cross-study comparisons remain difficult because RHF definitions vary by timing, severity, need for inotropes, need for RVAD support, and hemodynamic or laboratory thresholds.^8^, ^10^ This is especially relevant because early postoperative RHF, persistent RHF, and late RHF likely represent overlapping but clinically distinct entities. Registry data similarly demonstrate the frequency and prognostic importance of RHF after LVAD implantation. Kapelios et al. reported RHF in 24% of 5,537 continuous-flow LVAD recipients at 1 month, while Rame et al. showed that late RHF after LVAD implantation was associated with higher rates of complications and death.^2^, ^3^

In our cohort, the strongest association with RHF was increased resource utilization. Patients with RHF had longer ICU stays, total hospital stays, and post-implant hospitalizations than patients without RHF. This aligns with prior studies showing that RHF after LVAD implantation is associated with prolonged ventilation, longer ICU and hospital stays, end-organ dysfunction, and increased rehospitalization.^3^, ^11^ These findings reinforce that postoperative RHF is not only a survival-related complication but also a major driver of perioperative complexity and healthcare utilization.

### Temporary RV support strategies after LVAD implantation

Temporary RV support is considered when RHF is refractory to inotropes, pulmonary vasodilators, volume optimization, and LVAD speed adjustment. Current options include surgically implanted RVAD configurations, percutaneous dual-lumen systems such as ProtekDuo, extracorporeal RVAD strategies, and VA ECMO in patients with combined circulatory or pulmonary failure.^1^, ^5^, ^8^ However, support selection is rarely dictated by comparative evidence alone. Instead, it is shaped by institutional experience, timing of RHF recognition, patient acuity, respiratory status, and cannulation strategy.

Much of the contemporary literature has focused on ProtekDuo and VA ECMO. ProtekDuo offers internal jugular venous access, upper-body cannulation, potential mobility advantages, and bedside removal. Salna et al. reported 86% weaning from support and 85% survival to discharge among 27 patients receiving ProtekDuo after LVAD implantation.^6^ However, a systematic review by Alam et al. found wide variation in ProtekDuo outcomes, with in-hospital mortality ranging from 9% to 46% and weaning success from 24% to 91%.^12^ In a national database analysis, Beller et al. found that patients requiring RVAD support at LVAD implantation had lower operative mortality than those requiring VA ECMO, while delayed postoperative initiation of RV support was associated with worse outcomes regardless of modality.^13^ These findings suggest that timing, patient selection, and institutional pathways may be as important as the support device itself.

### T-VAD support and interpretation of outcomes

**Figure 3:**
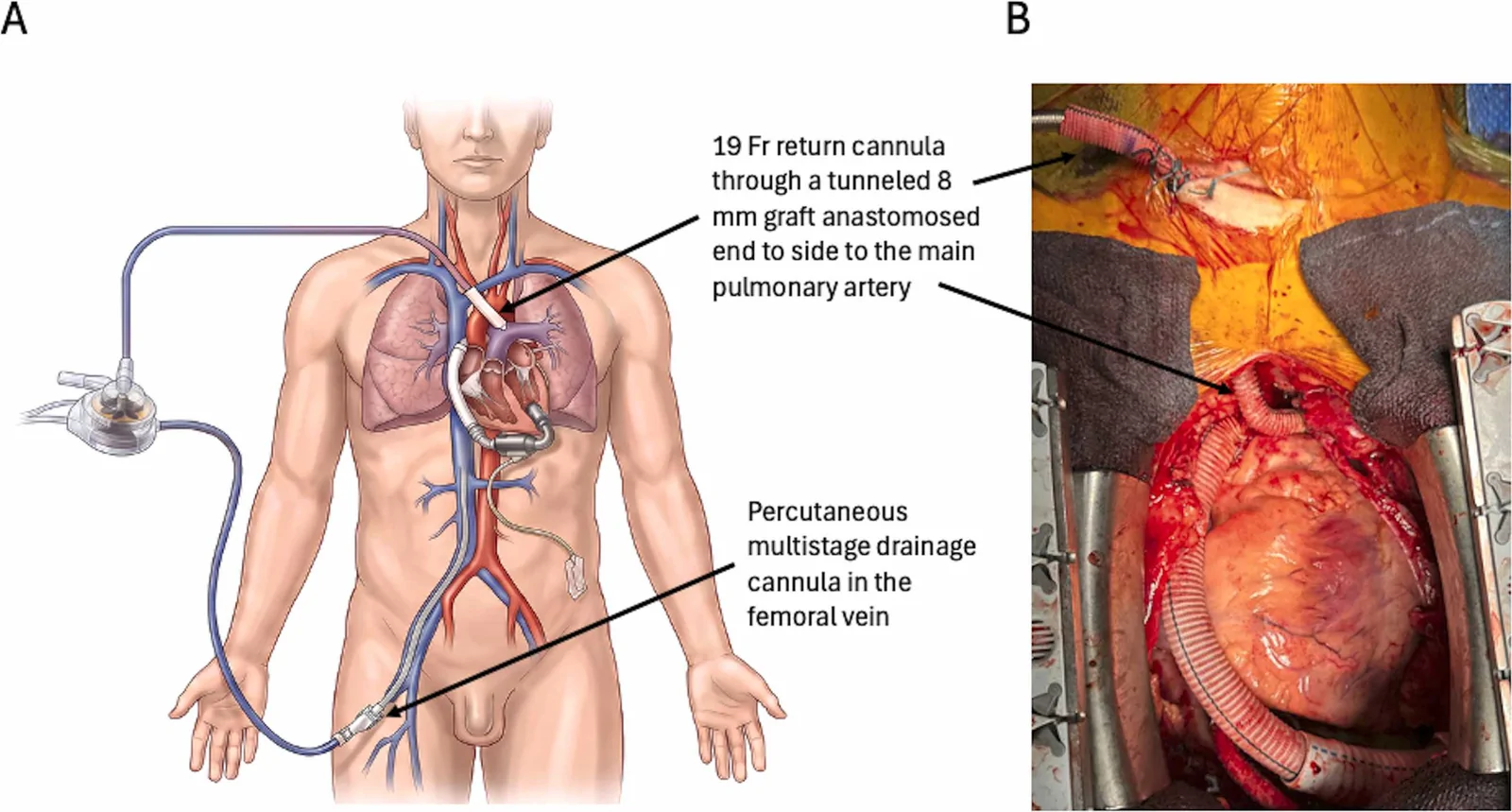
Surgical configuration of venous-to-pulmonary artery right ventricular support. Schematic illustration of the T-VAD configuration demonstrating percutaneous femoral venous drainage and return through a 19-Fr cannula positioned within an 8-mm graft anastomosed to the main pulmonary artery and tunneled to exit at the right side of the neck above the clavicle. (B) Intraoperative photograph demonstrating the tunneled graft and return cannula exiting at the right neck and its anastomosis to the main pulmonary artery. T-VAD, Tharakan-Ventricular Assist Device.

Our institutional approach primarily used T-VAD support, in which venous drainage is returned directly to the pulmonary artery through a graft-based configuration. This strategy differs from ProtekDuo and VA ECMO and should not be grouped interchangeably with other RV support modalities. Although ProtekDuo is often favored for upper-body cannulation and potential mobilization, T-VAD configuration allowed mobilization despite femoral venous drainage in selected patients. Graft-based venous-to-pulmonary artery support has been described previously, with grafts tunneled to subcostal or subxiphoid exit sites.^14^, ^15^ The T-VAD differs in that the graft exits at the right side of the neck, above the clavicle, rather than over the epigastrium, which does not restrict sitting or truncal flexion during support. Figure 3 illustrates the T-VAD support configuration, highlighting the tunneled pulmonary artery graft and return cannula exiting at the right side of the neck.

In this study, T-VAD refers specifically to our graft-based surgical configuration, whereas ProtekDuo was analyzed separately as a percutaneous dual-lumen support strategy. Potential advantages of T-VAD support include direct pulmonary artery return without traversing the tricuspid and pulmonary valves, reliable RV decompression, and maintaining adequate preload for the LVAD device. In selected patients with significant oxygenation needs, a membrane oxygenator may also be incorporated into the circuit, allowing the configuration to support both right-sided circulation and gas exchange when clinically necessary.

In this cohort, T-VAD support was accompanied by favorable observed survival, with 95% of T-VAD patients alive at follow-up. The one death in the T-VAD group was due to a neurological complication. We deliberately refrain from comparing this with the small rescue-support group, which differed in timing, indication, and likely severity.

The broader literature supports this cautious interpretation. Chamogeorgakis et al. showed that patients requiring temporary or durable RVAD support after LVAD implantation represent a high-risk population with poor survival.^16^ Similarly, Beller et al. emphasized that earlier RV support is associated with better outcomes than delayed escalation.^13^ Therefore, the favorable outcomes observed with T-VAD in this cohort may reflect not only the configuration itself, but also institutional experience, intraoperative decision-making, patient selection, and timing of deployment. Future studies should compare temporary RV support strategies using standardized RHF definitions, detailed hemodynamic data, oxygenator use, transfusion requirements, duration of support, and objective measures of RV-pulmonary artery coupling such as tricuspid annular plane systolic excursion to pulmonary artery systolic pressure ratio (TAPSE/PASP).

## Limitations

This study has several important limitations. First, as a retrospective single-center cohort, the findings may not be generalizable to other institutions with different patient populations, practice patterns, or device preferences. Denominators reflect available data; missing values were not imputed, and variables with substantial missingness should be interpreted accordingly. Second, RHF after LVAD implantation remains difficult to study because definitions vary across the literature with respect to timing, severity, hemodynamic criteria, inotrope duration, and need for temporary RV support. Our study relied on the postoperative RHF designation in the institutional dataset rather than a prospectively applied standardized definition, which may limit comparability with other studies. Finally, support strategy was determined primarily by era of implantation rather than patient-level criteria, with non–T-VAD strategies predominating earlier and T-VAD later. Observed differences across modalities are therefore confounded by calendar time, evolving institutional experience, and a learning curve.

## Conclusions

Postoperative RHF occurred in one-third of patients after durable LVAD implantation and was associated with significantly longer ICU, total hospital, and post-implant length of stay. T-VAD was the predominant temporary RV support strategy at our institution and was accompanied by favorable observed survival. However, given the small and heterogeneous comparator group, these findings should be considered hypothesis-generating rather than evidence of superiority over other support strategies. Future multicenter studies using standardized RHF definitions, detailed hemodynamic data, and objective measures of RV–pulmonary artery coupling are needed to better guide temporary RV support selection after LVAD implantation.

## Statement of ethics

This retrospective study was reviewed and approved by the University of Oklahoma Heart Institute Institutional Review Board, with a waiver of informed consent. All data were de-identified prior to analysis, and no identifiable clinical details, photographs, or patient-specific vignettes are included.

## Declaration of Competing Interest

The authors declare that they have no known competing financial interests or personal relationships that could have appeared to influence the work reported in this paper.

## Data Availability

The data supporting the findings of this study are not publicly available because they were derived from patient records and are subject to institutional and ethical restrictions. De-identified data may be made available from the corresponding author upon reasonable request and with appropriate institutional approval.
